# Prognostic and Predictive Value of *LIV1* Expression in Early Breast Cancer and by Molecular Subtype

**DOI:** 10.3390/pharmaceutics15030938

**Published:** 2023-03-14

**Authors:** Alexandre de Nonneville, Pascal Finetti, Laurys Boudin, Emilie Denicolaï, Daniel Birnbaum, Emilie Mamessier, François Bertucci

**Affiliations:** 1 Equipe labellisée « Ligue Nationale Contre le Cancer », Equipe ‘Predictive Oncology’, Centre de Recherche en Cancérologie de Marseille, Inserm, CNRS, Institut Paoli-Calmettes, Aix-Marseille University, 13009 Marseille, France; 2 Département d’Oncologie Médicale, Institut Paoli-Calmettes, 13009 Marseille, France

**Keywords:** LIV1, SLC39A6, ZIP6, breast cancer, antibody–drug conjugates, zinc transporter protein, expression, survival

## Abstract

Background: LIV1 is a transmembrane protein that may become a new therapeutic target through the development of antibody–drug conjugates (ADCs). Few studies are available regarding the assessment of *LIV1* expression in clinical breast cancer (BC) samples. Methods: We analyzed *LIV1* mRNA expression in 8982 primary BC. We searched for correlations between *LIV1* expression and clinicopathological data, including disease-free survival (DFS), overall survival (OS), pathological complete response to chemotherapy (pCR), and potential vulnerability and actionability to anti-cancer drugs used or under development in BC. Analyses were performed in the whole population and each molecular subtype separately. Results: LIV1 expression was associated with good-prognosis features and with longer DFS and OS in multivariate analysis. However, patients with high *LIV1* expression displayed a lower pCR rate than patients with low expression after anthracycline-based neoadjuvant chemotherapy, including in multivariate analysis adjusted on grade and molecular subtypes. *LIV1*-high tumors were associated with higher probabilities of sensitivity to hormone therapy and CDK4/6 inhibitors and lower probabilities of sensitivity to immune-checkpoint inhibitors and PARP inhibitors. These observations were different according to the molecular subtypes when analyzed separately. Conclusions: These results may provide novel insights into the clinical development and use of LIV1-targeted ADCs by identifying prognostic and predictive value of *LIV1* expression in each molecular subtype and associated vulnerability to other systemic therapies.

## 1. Introduction

Breast cancer (BC) is the most frequent cancer in women in Western countries and the leading cause of cancer death. Despite progress during recent decades [1], nearly 20% of BC patients still develop metastases and die from disease progression. Major therapeutic progress has been achieved thanks to the development of molecularly targeted therapies directed against oncogenic proteins (ERBB2, EGFR, VEGF, and PI3K/AKT/mTOR pathway), immune therapy directed against immune checkpoints (PD1 and PDL1), and, more recently, antibody–drug conjugates (ADCs). ADCs represent a major and fast-growing drug class [2]. They are composed of a monoclonal antibody (mAb) chemically linked (via the linker) to a cytotoxic drug, also known as the payload. Currently, three ADCs are approved in BC [3]. Trastuzumab emtansine in the HER2+ subtype [4], trastuzumab deruxtecan in the HER2+ [5] and HER2-low [6] subtypes, and sacituzumab govitecan in the triple-negative subtype (TN) [7] and recently the HR+/HER2- subtype [8]. Many others are under development, including our anti-nectin-4 ADC [9,10] and the ladiratuzumab vedotin anti-LIV1 ADC (SGN-LIV1A, Seagen; [11,12]).

LIV1 is a transmembrane zinc transporter protein (ZIP), which is typically found in hormonally regulated tissues, such as breast, where its expression is controlled by estrogens. The gene (*SLC39A6* or ZIP6, located on chromosome18q12.2) was originally identified in human BC cell lines as an mRNA induced by estrogen treatment [13,14]. This cell surface membrane protein was a promising candidate for ADC therapy due to its broad expression in several cancers, including BC, and limited normal tissue expression. The ZIP family transporters increase cytosolic zinc by regulating its influx from either extracellular or intracellular stores [15,16]. Given the important role of zinc in cellular metabolism and a multitude of cellular functions, and the involvement of zinc dysregulation in oncogenesis [17], expression of zinc transporters such as LIV1 has been studied in different cancers [18]: prostate, kidney, lung, pancreas, cervical, and head and neck and squamous cell carcinomas, with evidence of correlations with clinicopathological features in several studies. In BC, a preclinical study showed the promoter effect of LIV1 on epithelio–mesenchymal transition [19]. A few studies are available regarding the assessment of its mRNA and/or protein expression in clinical samples and analysis of correlations with clinicopathological variables [20,21,22,23]. Overall, they highlight the potential favorable prognostic value of LIV1 expression in early-stage BC, but none of them presented analyses per molecular subtype, nor explored the predictive value for response to chemotherapy or vulnerability/actionability to current or future systemic therapies of BC. This latter issue is all the most important in that LIV1 might become in the near future a new therapeutic target.

Here, we analyzed the *LIV1* mRNA expression in 8982 primary breast cancers. We searched for correlations with clinicopathological data, including disease-free survival, overall survival, pathological response to chemotherapy, and potential vulnerability and actionability to anti-cancer drugs used or under development in BC. Analyses were performed in the whole population and in each molecular subtype separately.

## 2. Materials and Methods

### 2.1. Breast Cancer Samples and Gene Expression Profiling

We analyzed our breast cancer gene expression database [24] pooled from 36 public datasets (Table S1), comprising 8982 non-redundant non-metastatic, non-inflammatory, primary, and invasive breast cancer samples, including 353 from our institution [25]. For each sample, both the gene expression profile generated using DNA microarrays or RNA-Seq and the clinicopathological annotations were available. These sets had been gathered from the National Center for Biotechnology Information (NCBI)/Genbank GEO, ArrayExpress, European Genome-Phenome Archive, the Cancer Genome Atlas portal (TCGA) databases, and authors’ website. The details of Institutional Review Board and Ethical Committee approval and patients’ consent for all 36 studies are present in their corresponding publications listed in Table S1.

### 2.2. Gene Expression Data Analysis

Several steps of pre-analytic data processing were applied. In the first step, each set was separately normalized. Normalization was performed in R using Bioconductor and associated packages; we used quantile normalization for the already processed data from non-Affymetrix-based sets (Agilent, SweGene, and Illumina), and Robust Multichip Average (RMA) with the non-parametric quantile algorithm for the raw Affymetrix data. In the second step, we mapped the hybridization probes across the different DNA microarrays represented as reported [26]. When multiple probes mapped to the same GeneID, we retained the most variant probe in a particular dataset. We log2-transformed the available TCGA RNA-Seq data that were already normalized. Before analysis, gene expression levels were standardized within each dataset, using the PAM50 luminal A population as a reference. This allowed exclusion of biases due to laboratory-specific variations and population heterogeneity, and to make data comparable across all sets. *LIV1/SLC39A6* expression in tumors was measured as a discrete value (high vs. low) by using the median expression level across the whole series as cut-off. The same discretization was applied for each gene analyzed individually. We also collected and analyzed the gene expression and protein expression data of 343 cancer cell lines, including 28 breast cell lines, of the Broad Institute Cancer Cell Line Encyclopedia (CCLE) [27] hosted on the Cancer Dependency Portal (DepMap). There was a significant positive correlation between the mRNA and protein expression levels of LIV1 (Figure S1) in all cell lines (r = 0.60, *p* = 2.52 × 10^−35^) and in breast cell lines (r = 0.70, *p* = 3.0 × 10^−5^), indirectly suggesting that the changes in gene expression in tumors likely translate into changes at the protein level for LIV1. To avoid biases related to trans-institutional IHC analyses, and thanks to the bimodal distribution of respective mRNA expression levels, the ER, progesterone receptor (PR), and HER2 statutes (negative/positive) were defined on transcriptional data of *ESR1*, *PGR*, and *HER2*, respectively, as described [28]. The molecular subtypes of samples were then defined as HR+ /HER2- for ER-positive and/or PR-positive and HER2-negative tumors, HER2+ for HER2-positive tumors, and triple-negative (TN) for ER-negative, PR-negative, and HER2-negative tumors. We also defined the LAR subtype and the non-LAR subtypes among the TN tumors according to the Lehmann’s signature [29]. Finally, and to assess the potential vulnerability or actionability of tumor samples to anti-cancer drugs used or in development in breast cancer, we applied to each dataset separately several multigene signatures: E2F4-activation signature predictive for response to hormone therapy [30], Rbsig signature predictive for resistance to CDK4/6 inhibitors [31], TIS (T-cell-inflamed signature) [32] predictive for response to immune checkpoint inhibitors (ICI), and the HRD signature [33] predictive for response to PARP inhibitors. We also extracted the expression levels of genes encoding for therapeutic targets of ADC (HER2, TACSTD2/TROP2, Nectin4). We also collected the DNA data from TCGA [34] (whole-exome sequencing (WES) and array-CGH) and METABRIC [35] (targeted-NGS, array-CGH) to assess the presence or absence of actionable genetic alterations of ESCAT I and II levels [36] in the tumors according to LIV1 expression status.

### 2.3. Statistical Analysis

Correlations between tumor classes and clinicopathological variables were analyzed using the Student’s *t*-test or Fisher’s exact test when appropriate. Disease-free survival (DFS) was calculated from the date of diagnosis until the date of relapse (local, regional, or distant) or death from any cause. Overall survival (OS) was calculated from the date of diagnosis until the date of death from any cause. Follow-up was measured from the date of diagnosis to the date of last news for event-free patients. Survivals were calculated using the Kaplan–Meier method and curves compared with the log-rank test. Univariate and multivariate prognostic analyses were performed using Cox regression analysis (Wald test). The variables submitted to univariate analyses included the LIV1-based classification and the classical prognostic factors of early-stage BC: patients’ age at diagnosis (>50 vs. ≤50 years), pathological axillary lymph node status (pN: positive vs. negative), pathological tumor size (pT2-pT3 vs. pT1), pathological grade (2–3 vs. 1), and the molecular subtypes (HER2+, TN vs. HR+/HER2-). We also analyzed the pathological complete response (pCR) rate after anthracycline-based neoadjuvant chemotherapy in the subset of 1203 patients who underwent neo-adjuvant chemotherapy: pCR was defined as the absence of invasive cancer in both breast and axillary lymph nodes on the operative specimen (ypT0/Tis ypN0; AJCC 8^th^ edition). Univariate and multivariate analyses for pCR were performed using logistic regression. The variables submitted to univariate analyses included the LIV1-based classification and patients’ age at diagnosis (>50 vs. ≤50 years), pathological grade (2–3 vs. 1), and the molecular subtypes (HER2+, TN vs. HR+/HER2-). The variables with a *p*-value < 0.05 in univariate analyses were tested in multivariate analyses. All statistical tests were two-sided at the 5% level of significance. Statistical analysis was performed using the survival package (version 2.30) in the R software (version 2.15.2; http://www.cran.r-project.org/ accessed on 14 December 2022). We followed the reporting REcommendations for tumor MARKer prognostic studies (REMARK criteria) [37].

## 3. Results

### 3.1. Patient Population and LIV1 Expression

LIV1 expression was analyzed in a total of 8982 invasive breast cancer samples. The clinicopathological characteristics are summarized in Table 1: overall, 64% of patients were >50 years old at diagnosis, 79% of tumors were ductal carcinoma, 47% were grade 3, 52% were pT2, 44% were pathologically node-positive, 66% were HR+/HER2-, 12% were HER2+, and 22% were TN. *LIV1* mRNA expression level was heterogeneous across all samples, with a range of intensities over six intervals in log_2_ scale (Figure S2).

### 3.2. Correlations of LIV1 Expression with Clinicopathological Features

Analysis was performed using LIV1 expression as a discrete variable (high vs. low). As shown in Table 1, high expression was associated (Fisher’s exact test) with good-prognosis features: patients’ age > 50 years (*p* = 4.28 × 10^−9^), lower pathological tumor grade (*p* = 2.12 × 10^−21^), smaller pathological tumor size (*p* = 4.42 × 10^−4^), positive ER status (*p* < 2.0 × 10^−255^), positive PR status (*p* < 2.0 × 10^−255^), negative HER2 status (*p* = 1.49 × 10^−58^), and HR+/HER2- subtype (*p* < 2.0 × 10^−255^). No correlation existed with the pathological axillary lymph node status.

### 3.3. Correlation with Disease-Free Survival

**Figure 1:**
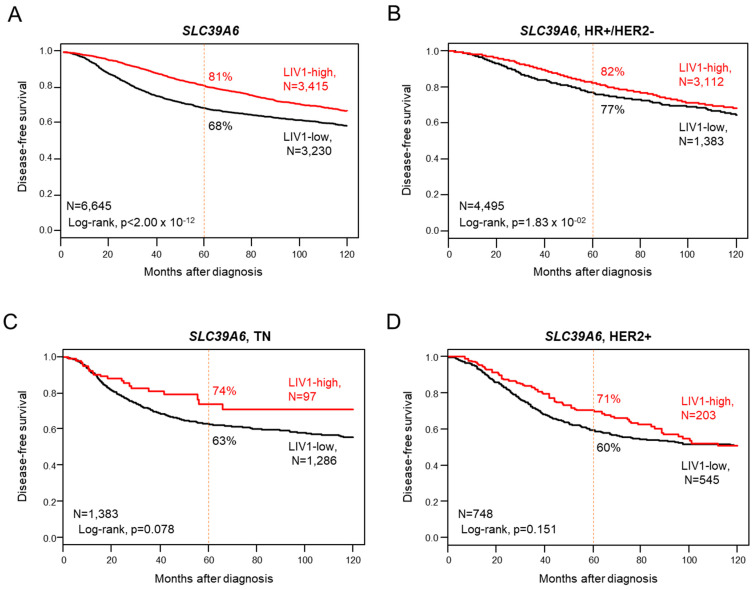
Prognostic value of LIV1 expression for DFS in breast cancer. Kaplan–Meier DFS curve according to high and low *LIV1* mRNA expression in the whole population (**A**), in the HR+/HER2- patients (**B**), in the TN patients (**C**), and in the HER2+ patients (**D**).

**Figure 2:**
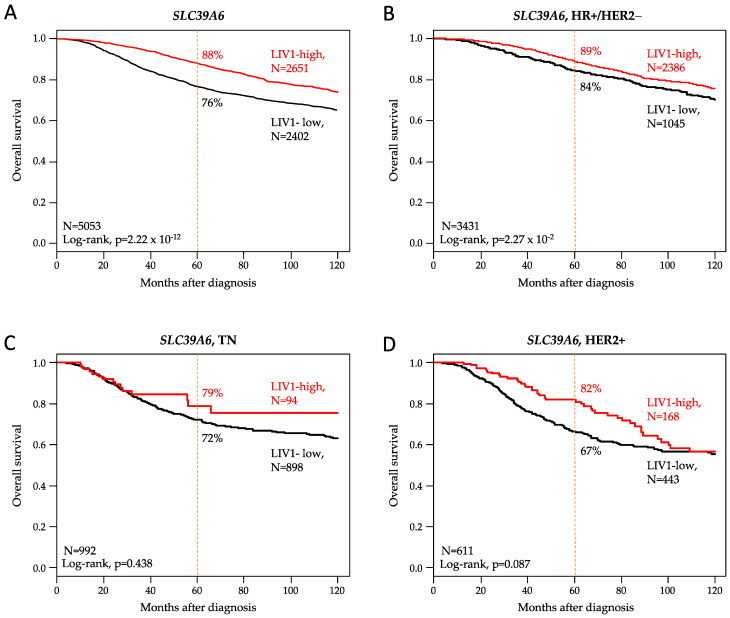
Prognostic value of LIV1 expression for OS in breast cancer. Kaplan–Meier OS curve according to high and low *LIV1* mRNA expression in the whole population (**A**), in the HR+/HER2- patients (**B**), in the TN patients (**C**), and in the HER2+ patients (**D**).

DFS data were available for 6645 patients: a total of 4754 (72%) remained event-free during a median follow-up of 68 months, 1891 (28%) displayed a DFS event, and the 5-year DFS was 75% (95% CI, 74–76). Patients with high LIV1 expression displayed longer 5-year DFS (81%, 95% CI 79–82) than those with low expression (68%, 95% CI 67–70; *p* < 2.0 × 10^−16^, log-rank test; Figure 1A and Figure 2).

The corresponding Hazard Ratio for a DFS event was 0.67 (95% CI 0.61–0.73). In univariate analysis (Table 2), patient’s age above 50 years was associated with longer DFS (*p* = 2.91 × 10^−4^, Wald test), whereas pathological grade 2–3 (*p* = 5.24 × 10^−14^), positive axillary lymph node status (*p* = 3.16 × 10^−19^), pT2-T3 tumor size (*p* = 1.12 × 10^−17^), and HER2+ and TN subtypes (*p* = 4.86 × 10^−35^) were associated with shorter DFS. In multivariate analysis, all the tested variables remained significant, including the LIV1 expression status (*p* = 3.88 × 10^−2^, Wald test), suggesting an independent prognostic value.

### 3.4. Correlation with Overall Survival

**Figure 3:**
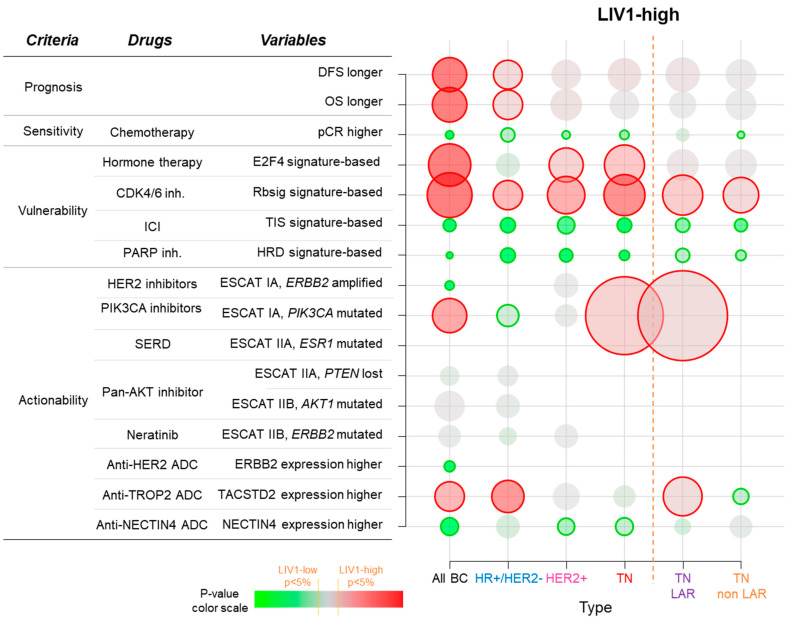
Correlations of LIV1 expression with clinical outcome and vulnerability/actionability to systemic treatments.

OS data were available for 5053 patients: a total of 3926 (78%) remained event-free during a median follow-up of 68 months, 1127 (22%) displayed an OS event, and the 5-year OS was 82% (95% CI, 81–84). Patients with high LIV1 expression displayed longer 5-year OS (88%, 95% CI 87–89) than those with low expression (76%, 95% CI 75–78; *p* = 2.22 × 10^−12^, log-rank test; Figure 2A and Figure 3).

The corresponding Hazard Ratio for an OS event was 0.66 (95% CI 0.58–0.74). In univariate analysis (Table 3), pathological grade 2–3 (*p* = 1.18 × 10^−14^), positive axillary lymph node status (*p* = 4.14 × 10^−31^), pT2-T3 tumor size (*p* = 9.81 × 10^−21^), and HER2+ and TN subtypes (*p* = 3.53 × 10^−23^) were associated with shorter OS. In multivariate analysis, all the tested variables remained significant, including the LIV1 expression status (*p* = 1.74 × 10^−3^, Wald test), suggesting an independent prognostic value.

### 3.5. Correlation with Pathological Response to Chemotherapy

The pathological response after neoadjuvant anthracycline-based chemotherapy was available for 1203 patients, with a 23% pCR rate (281/1203). In this sub-population, high LIV1 expression was associated (Fisher’s exact test, Table S2) with lower pathological tumor grade (*p* = 2.51 × 10^−5^), and HR+/HER2- subtype (*p* = 5.88 × 10^−71^). In univariate analysis (Table 4), the patients with high LIV1 expression displayed lower pCR rate than patients with low expression (Figure 3), with respective rates equal to 11% vs. 33% (*p* = 6.84 × 10^−20^, Fisher’s exact test) and an OR equal to 0.24 (95% CI 0.17–0.33, logit link function). Other variables associated with higher pCR rates were the pathological grade 2–3 (*p* = 3.93 × 10^−3^, logit link function) and the HER2+ (*p* = 1.12 × 10^−10^) and TN (*p* = 2.22 × 10^−15^) subtypes. In multivariate analysis, all tested variables remained significant, including LIV1 expression (*p* = 1.57 × 10^−7^), suggesting an independent predictive value.

The bubble heat map shows the correlation between high LIV1 expression and “favorable” variables with respect to (from top to bottom) prognosis (longer post-operative DFS and OS), sensitivity to neoadjuvant chemotherapy (higher pCR rate), vulnerability to hormone therapy (E2F4 signature), to CDK4/6 inhibitors (Rbsig signature), to ICI (TIS signature), and to PARP inhibitors (HRD signature), and actionability defined by high expression level of genes encoding for ADC targets (HER2, TACSTD2/TROP2, Nectin4) or by presence of actionable genetic alterations of ESCAT I level (HER2 amplification, PIK3CA mutations) and II level (ESR1 mutations, AKT mutations, PTEN deletion, and HER2 mutations). Analyses are shown in the whole series and in each BC subtype. The correlation with DFS and OS was assessed by Cox regression analysis and the correlations with other variables were assessed by logistic regression. A red bubble indicates a positive correlation (“favorable”), whereas a green bubble indicates a negative correlation (“unfavorable”), the color intensity indicates the statistical significance level (*p*-value) with a bold line encircling the bubble indicating significant correlation, and the size of the bubble indicates the statistical level.

### 3.6. Correlations with Potential Therapeutic Vulnerability or Actionability

We then assessed whether LIV1-high versus LIV1-low tumors displayed different potential vulnerability or actionability to anti-cancer drugs routinely used or in development in BC (Figure 3). Vulnerability was based on the status of multigene signatures associated with response or resistance. When compared to the LIV1-low tumors, the LIV1-high tumors were associated with higher probabilities of sensitivity to hormone therapy according to the E2F4-activation signature, [30] (*p* = 6.85 × 10^−68^) and of sensitivity to CDK4/6 inhibitors according to RBsig [31] (*p* = 9.07 × 10^−175^). By contrast, they displayed lower probabilities of sensitivity to ICI according to the TIS signature (*p* = 1.91 × 10^−197^) and of sensitivity to PARP inhibitors according to the HRD signature (*p* = 3.19 × 10^−279^). Actionability was based on the proportion of patients in each class displaying an actionable genomic alteration with a high evidence level according to the ESCAT scale [36]. LIV1-high tumors displayed a smaller proportion of cases with *ERBB2* amplification (*p* = 3.95 × 10^−10^), with HER2-high expression (*p* = 2.90 × 10^−53^), and with Nectin 4-high expression (*p* = 8.96 × 10^−23^), but a higher proportion of cases with *PIK3CA* mutation (*p* = 1.39 × 10^−6^), and with TROP2-high expression (*p* = 2.14 × 10^−5^) as compared to LIV1-low tumors. There was no significant difference regarding the ESCAT II level alterations (*ESR1* mutations, *PTEN* loss, *AKT1* mutations, *ERBB2* mutations).

### 3.7. Analysis of LIV1 Expression in HR+/HER2- Breast Cancers

We repeated these analyses in each molecular subtype separately. The HR+/HER2- subtype included 5929 tumor samples. LIV1 expression was not associated with the patient’s age or the pathological features (axillary lymph node status, tumor size, and grade; Table S3). Regarding DFS, 3380 out of 4495 informative patients (75%) remained event-free during the median follow-up of 72 months, and 1115 (25%) displayed a DFS event. The 5-year DFS rate was 80% (95% CI, 79–81). Here, too, the patients with high LIV1 expression displayed longer 5-year DFS (82%, 95% CI 80–83) than those with low expression (77%, 95% CI 75–79; *p* = 1.83 × 10^−2^, log-rank test; Figure 1B and Figure 3), and the corresponding Hazard Ratio was 0.86 (95% CI 0.76–0.98). In univariate analysis (Table S4), the pathological grade 2–3 (*p* = 4.56 × 10^−8^), positive axillary lymph node status (*p* = 9.24 × 10^−11^), and pT2-T3 tumor size (*p* = 1.58 × 10^−12^) were associated with shorter DFS. In multivariate analysis, all tested variables remained significant, including LIV1 expression (*p* = 2.57 × 10^−2^, Wald test). Similar results were observed in univariate and multivariate analyses for OS (Figure 2B and Figure 3, Table S4). Data concerning the pathological response to neoadjuvant chemotherapy were available for 575 patients, with a 12% pCR rate (70/575). In univariate analysis, high LIV1 expression was associated with a lower pCR rate than low expression (Figure 3), with respective rates equal to 9% vs. 19% (*p* = 3.11 × 10^−3^, Fisher’s exact test) and an OR equal to 0.45 (95% CI 0.27–0.75, *p* = 2.18 × 10^−3^, logit-link function). None of the other tested variables was significant (Table S5). When compared to LIV1-low tumors (Figure 2), the LIV1-high tumors displayed a higher probability of sensitivity to CDK4/6 inhibitors (*p* = 6.69 × 10^−5^), lower probability of sensitivity to ICI (*p* = 9.67 × 10^−64^), a lower proportion of cases with *PIK3CA* mutation (*p* = 2.64 × 10^−2^), and a higher proportion of cases with TROP2-high expression (*p* = 3.89 × 10^−8^).

### 3.8. Analysis of LIV1 Expression in TN Breast Cancers

Our series included 1936 TN tumor samples. High LIV1 expression was associated with positive axillary lymph node status (*p* = 4.36 × 10^−2^) and larger pathological tumor size pT2–3 (*p* = 6.29 × 10^−4^; Table S6). Regarding DFS, data were available in 1383 patients: the 5-year DFS rate was 64% (95% CI, 61–67), and high LIV1 expression tended to be associated with longer 5-year DFS (74%, 95% CI 64–86) than low expression (63%, 95% CI 60–66; *p* = 0.079, log-rank test; Figure 1C and Figure 3), with an HR equal to 0.69 (95% CI 0.45–1.05; *p* = 0.080, Wald test). In univariate analysis (Table S7), the pathological grade 2–3 (*p* = 3.43 × 10^−2^), positive axillary lymph node status (*p* = 4.81 × 10^−4^), and pT2-T3 tumor size (*p* = 7.27 × 10^−4^) were associated with shorter DFS. By contrast, LIV1 expression was not associated with OS (Figure 2C and Figure 3, Table S7). Data concerning the response to neoadjuvant chemotherapy were available for 467 patients, with a 33% pCR rate (155/467). In multivariate analysis, high LIV1 expression was associated with a lower pCR rate (Figure 2), with an OR equal to 0.28 (95% CI 0.14–0.56, *p* = 3.23 × 10^−4^, logit-link function; Table S8). When compared to LIV1-low tumors (Figure 3), the LIV1-high tumors displayed a higher probability of sensitivity to hormone therapy (*p* = 7.10 × 10^−4^) and CDK4/6 inhibitors (*p* = 1.15 × 10^−6^), lower probability of sensitivity to ICI (*p* = 8.33 × 10^−11^) and PARP inhibitors (*p* = 1.25 × 10^−10^), and a higher proportion of cases with *PIK3CA* mutation (*p* = 6.58 × 10^−3^), and a smaller proportion of cases with Nectin 4-high expression (*p* = 4.88 × 10^−4^). The same analysis was performed in the LAR subtype and the non-LAR subtypes separately. As shown in Figure 3, the LAR and non-LAR subtypes displayed very similar profiles, except more frequent ESCAT IA level *PIK3CA* mutations and TROP2-high expression in the LAR subtype than in the non-LAR subtypes.

### 3.9. Analysis of LIV1 Expression in HER2+ Breast Cancers

Our series included 1098 HER2+ tumor samples. LIV1 expression was not associated with patients’ age and pathological variables (Table S9). DFS data were available in 748 patients: the 5-year DFS rate was 60% (95% CI, 56–65), and LIV1 expression was not associated with DFS (*p* = 0.151; Figure 1D and Figure 3). High LIV1 expression tended to be associated with longer 5-year OS (82%, 95% CI 76–89) than low expression (67%, 95% CI 62–72; *p* = 0.087, log-rank test; Figure 2D and Figure 3), with an HR equal to 0.75 (95% CI 0.53–1.04; *p* = 0.088, Wald test), In univariate analysis (Table S10), the positive axillary lymph node status (*p* = 6.18 × 10^−4^), and pT2-T3 tumor size (*p* = 1.89 × 10^−7^) were associated with shorter OS. Data concerning the response to neoadjuvant chemotherapy were available for 161 patients, with a 35% pCR rate (56/161). In univariate analysis, high LIV1 expression was the sole significant variable and was associated with a lower pCR rate (Figure 3), with an OR equal to 0.24 (95% CI 0.11–0.53, *p* = 4.84 × 10^−4^, logit-link function; Table S11). When compared to LIV1-low tumors (Figure 3), the LIV1-high tumors displayed a higher probability of sensitivity to hormone therapy (*p* = 5.93 × 10^−4^) and CDK4/6 inhibitors (*p* = 9.42 × 10^−4^), lower probability of sensitivity to ICI (*p* = 1.84 × 10^−8^) and PARP inhibitors (*p* = 1.87 × 10^−10^), and a smaller proportion of cases with Nectin 4-high expression (*p* = 6.13 × 10^−3^).

## 4. Discussion

By examining *LIV1* mRNA expression in 8982 primary breast cancers, we found that *LIV1* expression was associated with good-prognosis features and favorable DFS and OS. However, patients with high *LIV1* expression displayed a lower pCR rate than patients with low expression after anthracycline-based neoadjuvant chemotherapy, including in multivariate analysis adjusted on grade and molecular subtypes. LIV1-high tumors were associated with higher probabilities of sensitivity to hormone therapy and CDK4/6 inhibitors and lower probabilities of sensitivity to ICI and PARP inhibitors.

Our analysis at the mRNA level allowed work on a very large series of BC samples and to search for associations with expression of clinically relevant signatures while avoiding the limitations of immunohistochemistry (standardization, reading subjectivity, cut-off). LIV1 expression was heterogeneous in BC samples, as reported at the protein and mRNA levels in published series. Few studies are available regarding the assessment of its mRNA and/or protein expression in clinical samples and analysis of correlations with clinicopathological variables [20,21,22,23]. Overall, they highlighted the potential favorable prognostic value of LIV1 expression in early-stage BC, but none of them presented analyses per molecular subtype, nor explored the predictive value for response to chemotherapy or vulnerability/actionability to current or future systemic therapies of BC. Kasper et al. [20] investigated LIV-1 expression pattern at mRNA and protein levels in 111 human breast cancers and found a negative correlation between protein expression and pathological tumor size and grade, as well as an association with longer relapse-free and overall survival. Liu et al. [21] analyzed data from ~1100 BC patients included in the TCGA database (241 basal, 611 luminal A, 431 luminal B, and 117 HER2-positive) and found an association of LIV1 mRNA expression with better OS in the whole cohort as well as in luminal A and HER2-positive patients. By contrast, OS was worse in LIV1-high patients in luminal B and TNBC patients. However, no multivariate analysis was performed. A study by Althobiti et al. [22] assessed *LIV1* mRNA expression and copy-number alterations using the METABRIC cohort (*n* = 1980) as well as immunohistochemistry in a large (*n* = 670) and annotated series of early-stage (I–III) operable BC. High *LIV1* mRNA was associated with ER-positivity, low grade, good Nottingham prognostic index (NPI), older age, and luminal A subtype. Longer BC-specific survival (BCSS) in all and ER-positive patients was observed in univariate and multivariate analyses. High LIV1 protein expression was associated with ER-positivity, low grade, low mitotic count, low nuclear pleomorphism, good NPI, and early nodal stage. Longer BCSS was observed in all and ER-positive patients in univariate analyses and was maintained in multivariate analysis only for ER-positive patients. Jones et al. [23] similarly identified a positive association between high LIV1 mRNA expression and outcome in 1879 and 4929 patients (for OS and relapse-free survival (RFS), respectively) using the publicly available online KmPlot database. However, no multivariate analysis was performed. LIV1 has been described as an estrogen-inducible gene that is upregulated in ER + BC and is part of the PAM50 signature (membrane permeability module) dedicated to prognostication of HR+/HER2- tumors [38,39]. Consistently, our results highlight the association between high *LIV1* expression and less aggressive tumors and a better outcome, especially in the ER-positive subgroup. High *LIV1* expression remained associated with longer DFS and OS and lower pCR rates in multivariate analysis independently from the molecular subtypes (and thus HR status). Thus, even if, from the point of view of ADC therapy, the key data are the abundance of target protein independently from the clinicopathological features, our correlation data suggest that HR+ tumors are more prone to be candidate to anti-LIV1 ADC than HR- tumors.

To our knowledge, no study had assessed the correlation between LIV1 expression in clinical samples and potential vulnerability or actionability to anti-cancer drugs used or in development in BC. Since LIV1-high tumors are likely more vulnerable to anti-LIV1 ADC than LIV1-low tumors, it is interesting to know their potential susceptibility to other current or future drugs. Our analyses were performed in the whole cohort as well as in each molecular subtype separately. In the whole cohort, 12 out of 16 tested variables were found as significant, but the results may be biased by subtypes. Thus, analysis per subtype was more relevant. In the HR+/HER2- subtype, LIV1-high tumors displayed a higher probability of sensitivity to CDK4/6 inhibitors, which represent the first-line treatment in advanced disease, a lower proportion of cases with *PIK3CA* mutation, the therapeutic target of alpelisib, a lower probability of sensitivity to ICI, and a higher proportion of cases with TROP2-high expression, candidates to sacituzumab govitecan ADC. In the TN subtype, they showed a lower probability of sensitivity to ICI and PARP inhibitors, a higher proportion of cases with *PIK3CA* mutation, and a smaller proportion of cases with Nectin 4-high expression. Finally, in the HER2+ subtype they displayed a higher probability of sensitivity to hormone therapy and CDK4/6 inhibitors, a lower probability of sensitivity to ICI and PARP inhibitors, and a smaller proportion of cases with Nectin 4-high expression. These results may provide novel insights into the clinical development and use of LIV1-targeted ADCs in the current therapeutic context. For example, they underline the need to enrich the therapeutical arsenal in LIV1-high TNBC that are at a lower probability to respond to ICI and PARP inhibitors that have recently challenged the clinical outcome at all stages of the disease. In the HR+/HER2- subtype, our data suggest, for example, that LIV1-low tumors might be more candidate to testing of ICI than LIV1-high tumors, suggesting “complementarity” between ICI and anti-LIV1 ADC. Combination of ADC with ICI are being tested: the potential lower sensitivity to ICI of LIV1-high tumors does not support interest for such a strategy with anti-LIV1 ADC. However, of course, these correlation data are hypothesis-generating, and warrant validation in pre-clinical models.

Ladiratuzumab vedotin is an ADC targeted against LIV1 conjugated by a protease-cleavable linker to the cytotoxic payload, a microtubule disruptor (monomethyl auristatin E). Promising clinical results were reported in metastatic BC. In a phase I clinical trial for patients with metastatic HR+/HER2- and TN BC (SGNLVA-001 trial: NCT01969643), out of 614 screened samples for LIV-1 status, 90% were positive, with moderate-to-high expression being detected in 82% of ER+/HER2- samples, 73% of HER2+, and 68% of TN. Patients with high or moderate expression were eligible and received at least two previous lines of therapy [40]. The updated results of this trial [41] showed a promising 28% objective response rate (ORR) in second-line refractory TNBC. Unfortunately, no results are available yet regarding ER-positive BC. In early-stage BC, ladiratuzumab vedotin was one of the experimental neoadjuvant treatments of the I-SPY2 trial (NCT01042379), combined with paclitaxel, administered for four cycles before doxorubicin/cyclophosphamide. This study arm was stopped due to reaching the predetermined time limit for patient accrual of 2 years. Sixty patients were randomized: ladiratuzumab vedotin did not improve pCR rates compared to the control arm [42]. In preclinical TNBC models, SGN-LIV1A induces an effective immunogenic cell death (ICD), potentially improving the benefit from ICI [43]. In order to boost the antitumor activity of ADCs, the combination of ICIs plus ladiratuzumab vedotin is under evaluation in two ongoing studies: one is exploring the combination with pembrolizumab as first-line treatment for metastatic TNBC (SGNLVA-002, Keynote-721, NCT03310957; phase Ib/II) with a promising preliminary ORR of 35% (66 patients), and the other one with atezolizumab as second-line treatment (Morpheus-TNBC, NCT03424005; phase Ib/II). Thus, the first two studies reported (SGNLVA-001 and I-SPY2) concerned HER2- patients: the results were negative for ISPY-2, encouraging for TN patients in SGNLVA-001 and not reported for HR+ patients in SGNLVA-001. Based on these preliminary results, the high clinical need of new drugs in TNBC, and pre-clinical data regarding the combination with ICI, the two ongoing studies were launched in metastatic TNBC in combination with immune therapy. This predominance of TNBC studies seems paradoxical given the higher expression of LIV1 in HR+ tumors as compared to TN tumors, which clearly justifies clinical evaluation of ladiratuzumab vedotin in HR+/HER2 BC.

## 5. Conclusions

We found that *LIV1* expression was associated with good-prognosis features and favorable survival, and with differential potential vulnerability or actionability to current drugs according to the molecular subtype. To our knowledge, this series is the largest one reported in BC. The main strength of our study lies in the number of samples analyzed allowing univariate and multivariate analyses per molecular subtype, and analysis of correlations with many clinically relevant features including expression of therapeutic targets and predictive signatures. Limitations include the retrospective nature and associated biases (missing ER, PR, and HER2 protein statutes for several samples leading to infer all statutes from the mRNA expression levels of corresponding genes), analysis of primary tumors and not metastatic samples, and the absence of analysis at the protein level. By highlighting the association of *LIV1* expression with clinical outcome and potential sensitivity, vulnerability, or actionability to drugs currently used or developed in BC, our results may provide novel insights into the clinical development and use of LIV1-targeted ADC.

## Figures and Tables

**Table 1:** Clinicopathological characteristics of the whole population and correlations with LIV1 expression status.

		N	All	LIV1 Class		
				Low	High	*p*-Value
Age at diagnosis (years)						4.28 × 10^−9^
	≤50	2540	2540 (36%)	1379 (40%)	1161 (33%)	
	>50	4488	4488 (64%)	2108 (60%)	2380 (67%)	
Pathological lymph node (pN)						0.469
	negative	3446	3446 (56%)	1668 (55%)	1778 (56%)	
	positive	2743	2743 (44%)	1354 (45%)	1389 (44%)	
Pathological tumor size (pT)						4.42 × 10^−4^
	pT1	2113	2113 (38%)	956 (35%)	1157 (40%)	
	pT2–3	3518	3518 (62%)	1763 (65%)	1755 (60%)	
Pathological tumor grade						2.12 × 10^−21^
	1	721	721 (11%)	246 (8%)	475 (15%)	
	2–3	5559	5559 (89%)	2946 (92%)	2613 (85%)	
ER status						<2.0 × 10^−255^
	negative	2764	2764 (31%)	2502 (56%)	262 (6%)	
	positive	6218	6218 (69%)	1989 (44%)	4229 (94%)	
PR status						<2.0 × 10^−255^
	negative	4670	4670 (52%)	3151 (71%)	1519 (34%)	
	positive	4255	4255 (48%)	1304 (29%)	2951 (66%)	
HER2 status						1.49 × 10^−58^
	negative	7884	7884 (88%)	3695 (82%)	4189 (93%)	
	positive	1098	1098 (12%)	796 (18%)	302 (7%)	
Molecular subtype						<2.0 × 10^−255^
	HR+/HER2-	5929	5929 (66%)	1878 (42%)	4051 (90%)	
	HER2+	1098	1098 (12%)	796 (18%)	302 (7%)	
	TN	1936	1936 (22%)	1801 (40%)	135 (3%)	
Pathological complete response (pCR)						6.84 × 10^−20^
	no pCR	922	922 (77%)	448 (67%)	474 (89%)	
	pCR	281	281 (23%)	224 (33%)	57 (11%)	
Follow-up median, months (min–max)		6645	68 (1–382)	62 (1–302)	68 (1–382)	6.42 × 10^−3^
DFS event, N (%)		6645	1891 (28%)	1072 (33%)	819 (24%)	1.17 × 10^−16^
5-year DFS (95% CI)		6645	75% (74–76)	68% (67–70)	81% (79–82)	<2.0 × 10^−16^
OS event, N (%)		5053	1127 (22%)	645 (27%)	482 (18%)	1.46 × 10^−13^
5-year OS (95% CI)		5053	82% (81–84)	76% (75–78)	88% (87–89)	2.22 × 10^−12^

**Table 2:** Univariate- and multivariate analyses for DFS in the whole population.

DFS	Univariate			Multivariate		
	N	HR (95% CI)	*p*-Value	N	HR (95% CI)	*p*-Value
Age at diagnosis (years), ≤50 vs. >50 years	5317	1.22 (1.10–1.37)	2.91 × 10^−4^	3229	1.25 (1.10–1.43)	7.37 × 10^−4^
Pathological lymph node (pN), pos vs. neg	5165	1.63 (1.47–1.82)	3.16 × 10^−19^	3229	1.46 (1.28–1.66)	9.64 × 10^−9^
Pathological tumour size (pT), pT2-pT3 vs. pT1	4719	1.68 (1.50–1.90)	1.12 × 10^−17^	3229	1.57 (1.37–1.80)	7.01 × 10^−11^
Pathological tumour grade, 2–3 vs. 1	4588	2.21 (1.80–2.72)	5.24 × 10^−14^	3229	1.58 (1.25–2.00)	1.30 × 10^−4^
Molecular subtype, HER2+ vs. HR+/HER2-	6626	1.86 (1.64–2.11)	4.86 × 10^−35^	3229	1.64 (1.37–1.98)	1.49 × 10^−7^
TN vs. HR+/HER2-		1.77 (1.59–1.97)		3229	1.25 (1.03–1.51)	2.21 × 10^−2^
LIV1 expression status, high vs. low	6645	0.67 (0.61–0.73)	4.87 × 10^−18^	3229	0.85 (0.73–0.99)	3.88 × 10^−2^

**Table 3:** Univariate and multivariate analyses for OS in the whole population.

DFS	Univariate			Multivariate		
	N	HR (95% CI)	*p*-Value	N	HR (95% CI)	*p*-Value
Age at diagnosis (years), ≤50 vs. >50 years	4542	1.05 (0.92–1.20}	0.440			
Pathological lymph node (pN), pos vs. neg	4274	2.22 (1.94–2.54}	4.14 × 10^−31^	3070	1.92 (1.66–2.22}	3.6 × 10^−18^
Pathological tumour size (pT), pT2-pT3 vs. pT1	4250	1.96 (1.70–2.26}	9.81 × 10^−21^	3070	1.68 (1.44–1.97}	3.68 × 10^−11^
Pathological tumour grade, 2–3 vs. 1	3745	2.98 (2.26–3.93}	1.18 × 10^−14^	3070	2.04 (1.51–2.76}	3.99 × 10^−6^
Molecular subtype, HER2+ vs. HR+/HER2-	5034	2.03 (1.73–2.38}	3.51 × 10^−23^	3070	1.66 (1.35–2.04}	1.13 × 10^−6^
TN vs. HR+/HER2-		1.70 (1.47–1.96}		3070	1.29 (1.05–1.58}	1.46 × 10^−2^
LIV1 expression status, high vs. low	5053	0.66 (0.58–0.74}	3.20 × 10^−12^	3070	0.76 (0.64–0.90}	1.74 × 10^−3^

**Table 4:** Univariate and multivariate analyses for pCR in the whole population.

pCR	Univariate			Multivariate		
	N	OR (95% CI)	*p*-Value	N	HR (95% CI)	*p*-Value
Age at diagnosis (years), ≤50 vs. >50 years	1202	1.16 (0.08–1.52)	0.262			
Pathological grade, 2–3 vs. 1	1097	8.07 (1.95–33.4)	3.93 × 10^−3^	1097	4.13 (0.98–17.5)	0.054
Molecular subtype, HER2+ vs. HR+/HER2-	1203	3.85 (2.55–5.79)	1.12 × 10^−10^	1097	2.77 (1.76–4.37)	1.21 × 10^−5^
TN vs. HR+/HER2-	1203	3.58 (2.61–4.91)	2.22 × 10^−15^	1097	2.31 (1.58–3.37)	1.37 × 10^−5^
LIV1 expression status, high vs. low	1203	0.24 (0.17–0.33)	1.65 × 10^−18^	1097	0.37 (0.25–0.54)	1.57 × 10^−5^

## Data Availability

All datasets are publicly available, and references are described in Table S1.

## Supplementary Materials

The following supporting information can be downloaded at: https://www.mdpi.com/article/10.3390/pharmaceutics15030938/s1, Figure S1: Correlations between mRNA and protein expression of LIV-1 in 343 cancer cell lines, including 28 breast cell lines, of the Broad Institute Cancer Cell Line Encyclopedia (CCLE). Figure S2: Distribution of *LIV1* mRNA expression levels (log_2_) across the 8982 breast cancer samples: violin plot and histogram. The red line represents the density curve of distribution. Table S1: List of breast cancer datasets included in the study; Table S2: Clinicopathological characteristics of the “pCR analysis” population and correlations with LIV1 expression status. Table S3: Clinicopathological characteristics of the HR+/HER2- tumors and correlations with LIV1 expression status. Table S4: Univariate and multivariate analyses for DFS and OS in the HR+/HER2- tumors; Table S5: Univariate analysis for pCR in the HR+/HER2- tumors; Table S6: Clinicopathological characteristics in the TN tumors and correlations with LIV1 expression status; Table S7: Univariate analysis for DFS and OS in the TN tumors; Table S8: Univariate and multivariate analyses for pCR in the TN tumors; Table S9: Clinicopathological characteristics in the HER2+ tumors and correlations with LIV1 expression status; Table S10: Univariate analyses for DFS and OS in the HER2+ tumors; Table S11: Univariate analysis for pCR in the HER2+ tumors.
