# Supplementary material for: Prognostic and Predictive Value of LIV1 Expression in Early Breast Cancer and by Molecular Subtype
Source: Pharmaceutics. 2023 Mar 14;15(3):938. doi: 10.3390/pharmaceutics15030938 (PMC10058875; doi:10.3390/pharmaceutics15030938)
Supplement: Supplementary file 1 [file pharmaceutics-15-00938-s001.zip › Figure S1.pptx]

## Slide 1
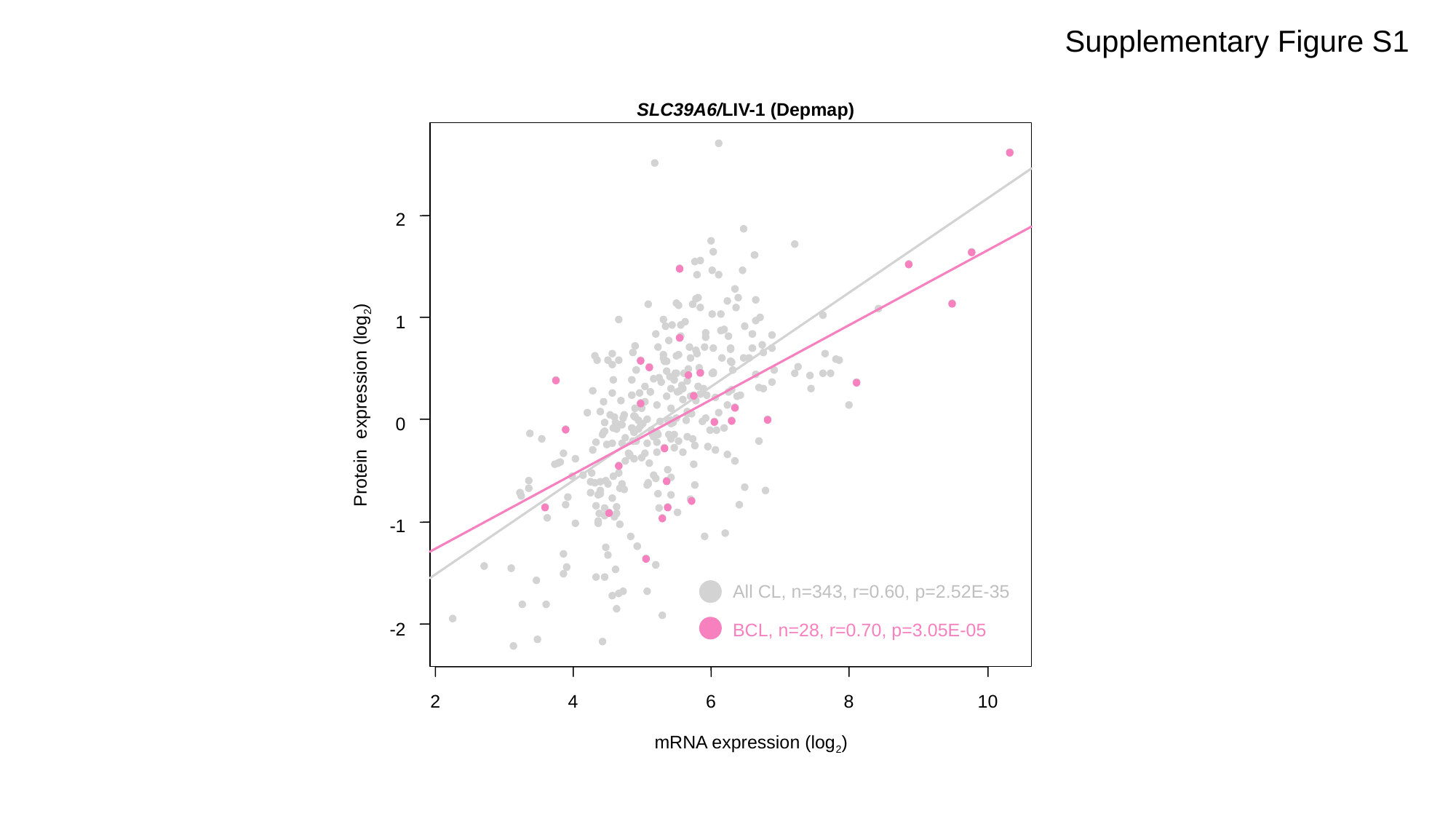

Supplementary Figure S1
SLC39A6/LIV-1 (Depmap)
Protein expression (log2)
All CL, n=343, r=0.60, p=2.52E-35
BCL, n=28, r=0.70, p=3.05E-05
mRNA expression (log2)
2
1
0
-1
-2
2
4
6
8
10
