# Supplementary figures and images for: Prognostic and Predictive Value of LIV1 Expression in Early Breast Cancer and by Molecular Subtype
Source: Pharmaceutics. 2023 Mar 14;15(3):938. doi: 10.3390/pharmaceutics15030938 (PMC10058875; doi:10.3390/pharmaceutics15030938)

## Slide 1
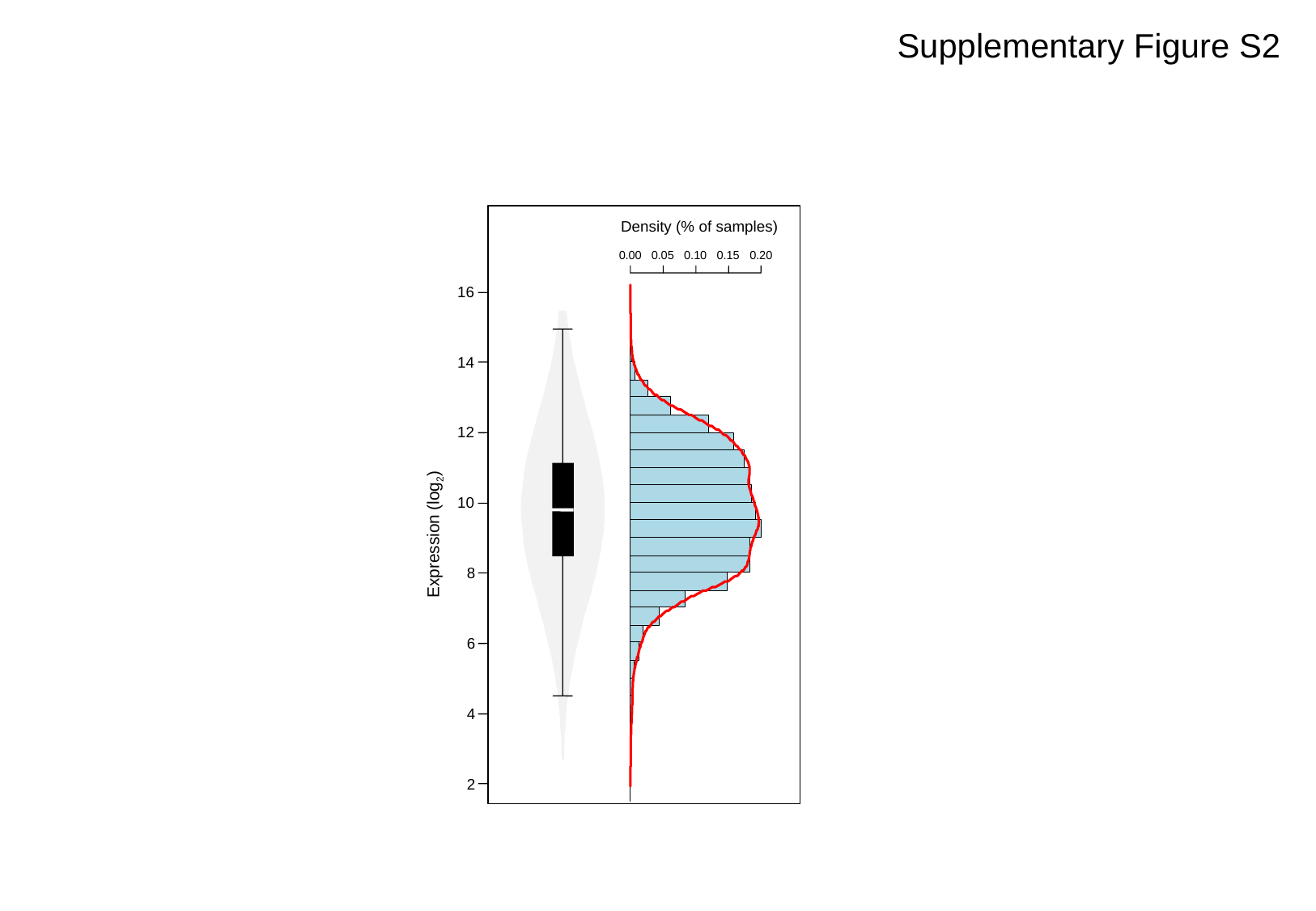

Supplementary Figure S2
Density (% of samples)
0.00
0.05
0.10
0.15
0.20
16
14
12
10
8
6
4
2
Expression (log2)

Supplement: Supplementary file 1 [file pharmaceutics-15-00938-s001.zip › Figure S2.pptx]
